# Impact of Different Childhood Adversities on 1-Year Outcomes of Psychotic Disorder in the Genetics and Psychosis Study

**DOI:** 10.1093/schbul/sbv131

**Published:** 2015-09-15

**Authors:** Antonella Trotta, Robin M. Murray, Anthony S. David, Anna Kolliakou, Jennifer O’Connor, Marta Di Forti, Paola Dazzan, Valeria Mondelli, Craig Morgan, Helen L. Fisher

**Affiliations:** ^1^Department of Psychosis Studies, Institute of Psychiatry, Psychology & Neuroscience, King’s College London, London, UK;; ^2^Department of Psychological Medicine, Institute of Psychiatry, Psychology & Neuroscience, King’s College London, London, UK;; ^3^MRC Social, Genetic and Developmental Psychiatry Centre, Institute of Psychiatry, Psychology & Neuroscience, King’s College London, London, UK;; ^4^Health Service and Population Research Department, Institute of Psychiatry, Psychology & Neuroscience, King’s College London, London, UK

**Keywords:** first episode, illness course, psychosis, trauma, psychotic symptoms, service use

## Abstract

While the role of childhood adversity in increasing the risk of psychosis has been extensively investigated, it is not clear what the impact of early adverse experiences is on the outcomes of psychotic disorders. Therefore, we investigated associations between childhood adversity and 1-year outcomes in 285 first-presentation psychosis patients. Exposure to childhood adversity prior to 17 years of age was assessed using the Childhood Experience of Care and Abuse Questionnaire. Data on illness course, symptom remission, length of psychiatric hospitalization, compliance with medication, employment, and relationship status were extracted from clinical records for the year following first contact with mental health services for psychosis. Seventy-one percent of patients reported exposure to at least 1 type of childhood adversity (physical abuse, sexual abuse, parental separation, parental death, disrupted family arrangements, or being taken into care). No robust associations were found between childhood adversity and illness course or remission. However, childhood physical abuse was associated with almost 3-fold increased odds of not being in a relationship at 1-year follow-up compared to patients who did not report such adverse experiences. There was also evidence of a significant association between parental separation in childhood and longer admissions to psychiatric wards during 1-year follow-up and 2-fold increased odds of noncompliance with medication compared to those not separated from their parents. Therefore, our findings suggest that there may be some specificity in the impact of childhood adversity on service use and social functioning among psychosis patients over the first year following presentation to mental health services.

## Introduction

There is a wealth of evidence suggestive of an association between childhood adversity (eg, physical and sexual abuse, neglect, death of or separation from a parent) and psychosis,^1–3^ reported from both general population^4–10^ and clinical studies.^11–14^ However, little is known about the effect of experiences of adversity during childhood on the course or outcomes of psychosis. There have been reports that childhood adversity is associated with persistence of psychotic symptoms,^15^ higher number of suicide attempts,^16^ poor medication adherence,^17^ and increased risk of readmission and relapse.^18^ In terms of social and vocational functioning, some previous studies have reported that childhood adversity is linked with a higher rate of unemployment^19^ and increased service costs,^20^ and these detrimental outcomes are maintained over time.^21^

However, other studies have not confirmed the effect of childhood adversity on clinical and social course of psychosis.^22,23^ The heterogeneity of the samples employed, the variety of outcome measures utilized, the reliance on self-rated assessments of adversity, and the tendency to focus on only 1 or 2 types of childhood adversity make it difficult to draw firm conclusions.

This study aimed to determine the impact of different types of childhood adversity on 1-year outcome across 3 domains (clinical, social, and service use) in a catchment-based sample of individuals presenting to mental health services for the first time with psychotic disorder. Consistent with previous studies that have shown that childhood adversity predicts an unfavorable course of depression^24^ and bipolar disorder,^16,25,26^ it was hypothesized that individuals with psychosis who reported exposure to any type of childhood adversity would have a worse outcome 1 year after first presentation when compared with those who did not report such adverse experiences.

## Methods

### Participants

The sample was drawn from patients who participated in the Genetics and Psychosis Biomedical Research Centre (GAP-BRC) study from the Lambeth, Southwark, Lewisham, and Croydon adult in-patient and out-patients units of the South London and Maudsley Mental Health NHS Foundation Trust (SLAM). Inclusion criteria for cases were: age 18–65 years, presenting to psychiatric services for the first time with a psychotic disorder (codes F20–29 and F30–33 from the International Classification of Diseases [ICD-10])^27^ and resident within tightly defined catchment areas in Southeast London, UK. Exclusion criteria were: organic psychosis, intelligence quotient (IQ) under 70, previous contact with services for psychosis, and transient psychotic symptoms resulting from acute intoxication. ICD-10 diagnoses were determined using data from the Schedules for Clinical Assessment in Neuropsychiatry (SCAN).^28^ Ethical permission was obtained from the SLAM and the Institute of Psychiatry Research Ethics Committee.

### Baseline Assessment

Baseline assessment and diagnostic interviews were performed by qualified psychologists and psychiatrists, subject to comprehensive training, and achievement of good interrater reliability. A range of sociodemographic information was obtained including age at interview, gender, current level of education, and self-ascribed ethnicity using the UK 2001 census categories. Duration of untreated psychosis (DUP) was defined as the period in weeks from the onset of psychosis to first contact with mental health services and it was calculated based on full clinical notes (with some informant interviews) of each patient using the Nottingham Onset Schedule-DUP.^29^ The Global Assessment of Functioning (GAF)^30^ scale was used to rate both severity of symptoms and disability. We assigned 2 separate ratings on the GAF based on dimensions of psychiatric symptoms (GAF-symptoms) and psychological, social, and occupational functioning (GAF-disability).

### Childhood Adversity

The Childhood Experience of Care and Abuse Questionnaire (CECA.Q)^31^ was employed at baseline to retrospectively elicit information from participants concerning a range of adverse childhood experiences. Physical abuse by the main mother and father figures (usually but not necessarily the biological parents), sexual abuse by an individual at least 5 years older than the recipient, separation from a parent for at least 6 months, death of a parent, taken into institutional care, and number of family arrangements, all prior to 17 years of age, were assessed. Every childhood experience section of the CECA.Q begins with screening questions and then positive responses are followed up with more detailed questions. The questionnaire was read out to all participants to improve the accuracy of the fixed category responses obtained. This questionnaire has been shown to have good internal consistency,^32^ satisfactory levels of test-retest reliability over 7 years in a similar psychosis sample,^33^ and reasonable concurrent validity with existing measures.^31–33^

### One-Year Follow-up

The 1-year follow-up period was taken as the date of first contact with SLAM mental health services for psychosis to the date exactly 1 year later using the clinical records held on the SLAM electronic Patient Journey System (ePJS). All of the following measures were completed by a researcher retrospectively from electronic mental health records.

### Follow-up Assessment and Definition of Outcomes

Information on course of illness (recovery, relapse episodes, continuous illness), remission from psychotic symptoms, relationship status, livelihood/occupation, compliance with medications, and number of days in hospital were collected with the Follow-up Psychiatric and Personal History Schedule (FU-PPHS).^34^ The FU-PPHS, previously used in World Health Organization multicenter studies of the incidence and outcome of schizophrenia^35^ and in previous studies of pathways to care,^36^ has shown good validity and reliability.^35^ Interrater reliability was established between 3 qualified psychologists on 10 training cases. Ratings of the presence or absence of symptoms were made on the basis of clear and definite information in the clinical records inserted by mental health professionals involved in patient care. Cohen’s κ values indicated robust agreement among the 3 raters (range: 0.583–1.000, *P* < .05). Efforts were made to maintain interrater reliability across the entire follow-up, including careful calibration and standardization procedures and regular, in-depth review of a sample of assessments. Raters were blind to diagnostic information from previous baseline assessments.

Relapse was defined as the emergence of positive, negative, or disorganized symptoms following a period of remission of at least 30 days. Similarly, “remission” was also operationally defined as the absence of such symptoms for at least 30 days. Noncompliance with medications was defined as: “lapses of 3 or more days more than once” or “not taking any prescribed medication.” The total number of days spent in institutions included in-patient treatment in a psychiatric hospital ward to which the patient had been admitted because of a psychiatric disorder. Relationship status was defined as married or in a steady relationship at follow-up vs being single, divorced, or widowed at follow-up. Employment status was defined as being employed or involved in a study program in the last 30 days of follow-up vs being unemployed or not studying at follow-up. Student status was defined as being a full-time student at secondary school, technical or occupational college, or University.

GAF^30^ scales were also completed from clinical records for the 7 days prior to the 1-year anniversary of first contact with mental health services for psychosis. Three researchers involved in rating the GAF via notes completed intensive reliability checks (intraclass correlation range: 0.974–1.000, all *P*’s < .001). The same raters were involved in the GAF and FU-PPHS record-based assessments to improve reliability.

### Statistical Analysis

The guidelines published by Bifulco et al^31^ were employed to create dichotomous variables for all the CECA.Q subscales. A composite variable was also computed to summarize how many of the different adversities had been experienced by each individual. This “total adversity” score involved summing the dichotomous CECA.Q subscale scores (range 0–6) and then recoding the total into an ordinal scale of 0 (none), 1 (single adverse experience), and 2 (multiple adverse experiences).

The total number of days spent in an institution for psychosis throughout the year following the first contact with mental health services was counted up (range 0–365 d). As the number of admission days was nonnormally distributed, with skewness of 1.71 (SE = 0.16) and kurtosis of 3.42 (SE = 0.32), the number of days that patients spent on a psychiatric ward was dichotomized at the median into less than 49 days vs 49 days or more.

Binary logistic regressions were used to analyze the relationship between each form of adversity and dichotomous follow-up variables (symptomatic remission, length of hospital admission, compliance with medications, relationship and employment status). Ordered logistic regressions and linear regressions were used for ordinal (illness course) and continuous normally distributed follow-up outcome variables (GAF-symptoms and GAF-disability scores at 1 year), respectively. All analyses were conducted using STATA release 11 (Stata-Corp).

## Results

### Prevalence of Childhood Adversity

Information on childhood adversity was available for 285 first-presentation psychosis patients. Exposure to at least 1 type of childhood adversity was found in 203 patients (71.2%), with 82 (28.8%) reporting multiple exposures. The most frequently occurring adverse childhood events were separation from parents (56.5%, *n* = 160), followed by physical abuse (22.8%, *n* = 65), disrupted family arrangements (3 or more arrangements; 20.7%, *n* = 56), and sexual abuse (14.4%, *n* = 41). Very few participants in this sample reported parental loss (11.7%, *n* = 33) or being taken into care during childhood (4.9%, *n* = 14). We have previously shown that all types of childhood adversity, except for sexual abuse, occurred more often among psychosis cases than unaffected controls.^11^ Patients reporting childhood adversity had a lower level of education (*P* < .001), were more often of non-White ethnicity (*P* < .001), and had lower GAF-disability scores at baseline (*P* = .033) compared with patients who did not (see table 1). These variables were controlled for in the final adjusted model, where appropriate.

**Table 1.** Baseline Sociodemographic and Clinical Characteristics of First-Presentation Psychosis Patients

		Any Childhood Adversity				
Demographic and Clinical Characteristics	Total (*N* = 285) *n* (%)	Yes (*N* = 203) *n* (%)	No (*N* = 82) *n* (%)	χ^2^	***df***	***P***
Gender				1.44	1	.285
Men	172 (60.4)	127 (62.6)	45 (54.9)			
Women	113 (39.6)	76 (37.4)	37 (45.1)			
Ethnicity				**36.28**	**5**	**< .001**
White British	72 (25.3)	39 (19.2)	33 (40.2)			
Black Caribbean	56 (19.6)	52 (25.6)	4 (4.9)			
Black African	65 (22.8)	50 (24.6)	15 (18.3)			
White other	30 (10.5)	21 (10.3)	9 (11.0)			
Asian (all)	24 (8.4)	10 (4.9)	14 (17.1)			
Other	38 (13.3)	31 (15.3)	7 (8.5)			
Level of education				**20.43**	**4**	**< .001**
No qualifications	48 (17.6)	40 (20.7)	8 (10.1)			
GCSE/O level	64 (23.5)	47 (24.4)	17 (21.5)			
A level	40 (14.7)	25 (13.0)	15 (19.0)			
Vocational/college	66 (24.3)	54 (28.0)	12 (15.2)			
University or professional qualifications	54 (19.9)	27 (14.0)	27 (34.2)			
Age (y)				*t* = 0.934	282	.351
Mean (SD)	28.9 (9.3)	28.6 (8.8)	29.7 (10.2)			
Duration of untreated psychosis (wk)				*t* = −0.773	173	.440
Mean (SD)	6.8 (11.0)	7.3 (11.6)	5.9 (9.8)			
GAF-symptoms score				*t* = 1.80	139	.073
Mean (SD)	46.9 (18.8)	44.9 (18.7)	51.4 (21.7)			
GAF-disability score				***t* = 2.16**	**138**	**.033**
Mean (SD)	55.7 (16.9)	53.7 (15.6)	60.3 (18.9)			

*Note:* Figures in bold indicate *P* <.05. Any childhood adversity refers to reported exposure prior to 17 y of age to any of the following: separation from a parent, parental death, physical abuse, sexual abuse, being taken into care, or having more than 2 family arrangements. *df*, Degrees of freedom; GAF, Global Assessment of Functioning scale; GCSE, General Certificate of Secondary Education.

### Follow-up Attrition Rate

At follow-up, of the 285 patients initially identified, 3 had died, 11 had left the country, 12 were discharged to a general practitioner (*n* = 6) or other mental health services (*n* = 6), and 22 were excluded on the basis of insufficient information available on ePJS. A total of 237 psychosis cases were active in ePJS at point of follow-up, giving a completion rate of 83.2%.

When patients with follow-up information available were compared with those without, there was a trend for difference in terms of gender (χ^2^ = 2.584, *P* = .145), but no evidence of systematic differences by age (*t* = −0.247, *P* = .805), ethnicity (χ^2^ = 10.673, *P* = .470), educational attainment (χ^2^ = 5.584, *P* = .235), baseline relationship status (χ^2^ = 0.349, *P* = .693), employment status (χ^2^ = 0.004, *P* = 1.000), or GAF-disability score (*t* = −0.828, *P* = .409). Similarly, patients with follow-up data did not differ in terms of clinical functioning at baseline (DUP *t* = 1.146, *P* = .253; GAF-symptom score *t* = −0.724; 0.470) or diagnosis (χ^2^ = 1.104, *P* = .622) from those without data. Additionally, there was no evidence that those who were not traceable were more likely to report a history of parental separation (χ^2^ = 1.005, *P* = .341), parental loss (χ^2^ = 0.540, *P* = .621), physical abuse (χ^2^ = 2.217, *P* = .186), sexual abuse (χ^2^ = 1.717, *P* = .260), institutional care (χ^2^ = 0.069, *P* = 1.000), or disrupted family arrangements (χ^2^ = 0.197, *P* = .693).

### Childhood Adversity and Clinical Course of Psychosis

Over the first year of contact with mental health services, a total of 123 (55.1%) patients had no relapse episodes of psychotic symptoms following their initial episode. There was no evidence of associations between each type of childhood adversity with course of psychosis over the 1-year follow-up period (table 2).

**Table 2.** Adjusted Associations Between Different Types of Childhood Adversity and 1-y Clinical Outcomes

	Course of Illness					Remission			
Type of Childhood Adversity	No Relapses, Complete or Nearly Complete Recovery *n* (%)	One or More Relapses *n* (%)	Continuous Illness *n* (%)	OR^a^ (95% CI)	*P*	Yes *n* (%)	No *n* (%)	OR^a^ (95% CI)	*P*
Parental separation									
No (*n* = 97)	56 (57.7)	21 (21.6)	20 (20.6)	-	-	66 (67.3)	32 (32.6)	-	-
Yes (*n* = 128)	68 (53.1)	29 (22.7)	31 (24.2)	1.25 (0.56–2.78)	.583	88 (67.2)	33 (32.8)	0.99 (0.42–2.33)	.982
Parental loss									
No (*n* = 200)	108 (54.0)	48 (24.0)	44 (22.0)	—	—	136 (67.3)	66 (32.7)	—	—
Yes (*n* = 24)	15 (62.5)	2 (8.3)	7 (29.2)	0.39 (0.10–1.58)	.187	17 (65.4)	9 (34.6)	1.84 (0.49–6.77)	.361
Physical abuse									
No (*n* = 176)	101 (57.4)	38 (21.6)	37 (21.0)	—	—	119 (67.6)	57 (32.4)	—	—
Yes (*n* = 51)	24 (47.7)	12 (23.5)	15 (29.4)	1.02 (0.36–2.86)	.973	36 (65.4)	19 (34.5)	1.00 (0.35–2.90)	.998
Sexual abuse									
No (*n* = 193)	105 (54.4)	43 (22.3)	45 (23.3)	—	—	131 (66.8)	65 (33.2)	—	—
Yes (*n* = 34)	20 (58.8)	7 (20.6)	7 (20.6)	1.06	.918	24 (68.6)	11 (31.4)	0.87	.819
				(0.33–3.39)				(0.26–2.92)	
Institutional care									
No (*n* = 216)	116 (53.70)	49 (22.69)	51 (23.61)	—	—	146 (66.4)	74 (33.6)	—	—
Yes (*n* = 11)	9 (81.82)	1 (9.09)	1 (9.09)	0.43	.473	9 (81.8)	2 (18.2)	2.50	.437
				(0.04–4.43)				(0.25–25.06)	
Family arrangements									
Up to 2 (*n* = 173)	98 (56.65)	35 (20.23)	40 (23.12)	—	—	116 (66.7)	58 (33.3)	—	—
3 or more (*n* = 46)	23 (50.00)	12 (26.09)	11 (23.91)	0.74	.558	31 (64.6)	17 (35.4)	1.92	.256
				(0.27–2.02)				(0.62–5.94)	
Total adversity									
0 (*n* = 65)	39 (60.0)	16 (24.6)	10 (15.4)	—	—	44 (67.7)	21 (32.3)	—	—
1 (*n* = 92)	46 (50.0)	20 (21.7)	26 (28.3)	1.09	.847	61 (65.6)	32 (34.4)	1.53	.390
				(0.45–2.65)				(0.60–4.04)	
2 or more (*n* = 70)	40 (57.1)	14 (20.0)	16 (22.9)	0.77	.632	50 (68.5)	23 (32.5)	1.51	.468
				(0.27–2.20)				(0.50–4.57)	

^a^Adjusted for duration of untreated psychosis and baseline Global Assessment of Functioning symptoms score.

A total of 155 patients (67.1%) had a period of at least 30 days without psychotic symptoms during the first year of contact with mental health services. However, more than half the sample (55%, *n* = 138) reported moderate or severe symptoms 1 year after their first presentation to services (GAF-symptoms < 61). No evidence of associations were found between childhood adversity and either remission from psychotic symptoms (table 2) or for GAF-symptom scores at 1-year follow-up (table 3), except for parental loss which was strongly associated with lower symptom levels at 1 year (*P* = .003) and the association remained significant when a Bonferroni correction for multiple testing was applied (*P* = .05/8 = .006). There was no robust evidence of a dose-response effect for exposure to multiple adverse experiences on clinical course of psychosis, symptomatic remission, or global clinical functioning over 1-year follow-up in this sample.

**Table 3.** Adjusted Associations Between Different Types of Childhood Adversity and Overall Clinical and Social Functioning at 1-y Follow-up

Type of Childhood Adversity	GAF Symptoms Mean (SD)	B^a^ (95% CI)	*P*	GAF Disability Mean (SD)	B^b^ (95% CI)	*P*
Parental separation						
No (*n* = 93)	59.3 (18.63)	—	—	54.7 (19.49)	—	—
Yes (*n* = 119)	58.0 (20.63)	−7.14 (−15.49 to 1.20)	.092	55.3 (19.71)	−1.12 (−9.48 to 7.25)	.791
Parental loss						
No (*n* = 187)	58.0 (19.17)	—	—	54.4 (18.82)	—	—
Yes (*n* = 24)	60.2 (24.22)	**18.72** (6.60–30.84)	**.003**	59.6 (24.15)	**22.64** (11.03–34.28)	**<.001**
Physical abuse						
No (*n* = 164)	58.7 (19.36)	—	—	55.3 (19.56)	—	—
Yes (*n* = 50)	57.7 (28.94)	−1.98 (−12.47 to 8.51)	.708	54.4 (19.50)	−0.46 (−11.04 to 10.12)	.931
Sexual abuse						
No (*n* = 181)	58.4 (19.84)	—	—	55.3 (19.38)	—	—
Yes (*n* = 33)	58.8 (19.19)	−0.81 (−12.84 to 11.21)	.893	54.1 (20.46)	−3.41 (−15.10 to 8.27)	.563
Institutional care						
No (*n* = 204)	58.1 (19.68)	—	—	54.5 (19.41)	—	—
Yes (*n* = 10)	67.0 (18.89)	10.83 (−9.28 to 30.95)	.288	67.3 (18.35)	12.42 (−7.33 to 32.17)	.215
Family arrangements						
Up to 2 (*n* = 162)	59.0 (19.71)	—	—	54.93 (19.48)	—	—
3 or more (*n* = 43)	55.8 (20.52)	1.30 (−9.68 to 12.29)	.814	56.42 (20.47)	3.63 (−7.21 to 14.46)	.508
Total adversity						
0 (*n* = 62)	59.6 (17.72)	—	—	55.4 (19.7)	—	—
1 (*n* = 85)	57.5 (19.80)	−2.31 (−12.02 to 7.38)	.636	53.5 (18.0)	0.41 (−9.42 to 10.23)	.935
2 or more (*n* = 67)	58.8 (21.40)	2.71 (−8.49 to 13.91)	.632	56.8 (21.3)	6.67 (−4.33 to 17.68)	.231

*Note: B*, regression coefficient. Figures in bold indicate *P* < .05.

^a^Adjusted for duration of untreated psychosis and baseline Global Assessment of Functioning (GAF) symptom score.

^b^Adjusted for duration of untreated psychosis and baseline Global Assessment of Functioning (GAF) disability score.

### Childhood Adversity and Social Outcomes of Psychosis

Table 4 presents the associations between types of childhood adversity and 1-year social outcomes. After adjustment for relationship status at baseline, reported exposure to physical abuse was associated with not being in a relationship at follow-up (*P* = .035), with almost a 3-fold increase in odds compared to patients who did not report this type of adversity (OR = 2.82). No associations were evident for the other adversities. A total of 169 (75.1%) patients of the overall sample were unemployed or not studying at 1 year. There was no evidence of associations with unemployment status at 1-year follow-up for psychosis cases reporting a history of childhood adversity compared to those who did not and no evidence of a dose-response effect for repeated adversity exposure.

**Table 4.** Adjusted Associations Between Different Types of Childhood Adversity and 1-y Social Outcomes

	Relationship Status				Employment Status			
Type of Childhood Adversity	In a Relationship *n* (%)	Not in a Relationship *n* (%)	OR^a^ (95% CI)	*P*	Employed *n* (%)	Not Employed *n* (%)	OR^b^ (95% CI)	*P*
Parental separation								
No (*n* = 100)	33 (33.0)	67 (67.0)	—	—	27 (28.1)	69 (71.9)	-	-
Yes (*n* = 131)	34 (25.9)	97 (74.1)	1.16 (0.56–2.39)	.655	28 (22.0)	99 (78.0)	0.97 (0.48–1.96)	.932
Parental loss								
No (*n* = 207)	61 (29.5)	146 (70.5)	—	—	47 (23.4)	154 (76.6)	-	-
Yes (*n* = 23)	7 (30.4)	16 (69.6)	0.84 (0.27–2.63)	.765	8 (38.1)	13 (61.9)	0.52 (1.17–1.53)	.234
Physical abuse								
No (*n* = 179)	56 (31.3)	123 (68.7)	—	—	47 (26.6)	130 (73.4)	-	-
Yes (*n* = 54)	12 (22.2)	42 (77.8)	**2.82** (1.07–7.43)	**.035**	9 (18.7)	39 (81.3)	1.67 (0.67–4.17)	.273
Sexual abuse								
No (*n* = 199)	61 (30.7)	138 (69.3)	—	—	48 (25.1)	143 (74.9)	-	-
Yes (*n* = 34)	7 (20.6)	27 (79.4)	1.33 (0.48–3.74)	.583	8 (23.5)	26 (76.5)	0.96 (0.37–2.49)	.928
Institutional care								
No (*n* = 222)	66 (29.7)	156 (70.3)	—	—	52 (24.3)	162 (75.7)	-	-
Yes (*n* = 11)	2 (18.2)	9 (81.8)	1.79 (0.30–10.44)	.521	4 (36.4)	7 (63.6)	0.47 (0.11–1.99)	.304
Family arrangements								
Up to 2 (*n* = 179)	52 (29.1)	127 (70.9)	—	—	48 (27.6)	126 (72.4)	-	-
3 or more (*n* = 45)	15 (33.3)	30 (66.7)	0.85 (0.34–2.07)	.716	8 (19.0)	34 (81.0)	1.54 (0.60–3.92)	.365
Total adversity								
0 (*n* = 67)	22 (32.8)	45 (67.2)	—	—	20 (30.3)	46 (69.7)	-	-
1 (*n* = 96)	30 (31.2)	66 (68.8)	0.93 (0.40–2.13)	.861	21 (22.6)	72 (77.4)	0.84 (0.36–1.95)	.687
2 or more (*n* = 70)	16 (22.9)	54 (77.1)	1.56 (0.61–3.99)	.348	15 (22.7)	51 (77.3)	1.14 (0.47–2.75)	.774

*Note*: Figures in bold indicate *P* < .05.

^a^Adjusted for baseline relationship status

^b^Adjusted for baseline employment status.

In terms of overall social functioning at 1 year, a total of 169 (67.3%) patients showed moderate or severe disability 1 year after first presentation to services (GAF-disability < 61). There was no evidence of associations with GAF-disability scores for most types of adversity (table 3), though there was evidence that experiences of parental loss were associated with better functioning at 1 year (*P* < .001), also after correcting for multiple testing (*P* = .05/8 = .006). However, the CI were very wide (11.03–34.28) indicating that this result should be interpreted cautiously. No robust evidence of a cumulative effect for repeated adverse experiences on social outcomes of psychosis over 1-year follow-up was found.

### Childhood Adversity and Service Use

The median length of admission over the first year since presentation to services was 48.5 days spent in hospital (interquartile range 21–102 days) and 64.1% of the sample (*n* = 132) was compliant with prescribed medications at 1-year follow-up. Results of the association between childhood adversity and length of hospitalization and medication compliance are shown in table 5. There was evidence of an association between parental separation in childhood and a longer admission to a psychiatric ward during 1-year follow-up, with cases reporting such adversity being approximately twice as likely to have longer hospital stays compared to those without such a history (*P* = .012). The association with length of hospitalization was stronger for participants who reported multiple (OR = 2.18, 95% CI: 1.11–4.29, *P* = .023) than single (OR = 1.57, 95% CI: 0.83–2.97, *P* = .164) adverse childhood experiences. A score test for trend provided evidence for a linear trend (*z* = 2.27, *P* = .023), indicating a dose-response effect on length of hospitalization for repeated adverse experiences.

**Table 5.** Adjusted Associations Between Different Types of Childhood Adversity and 1-y Service Use

	Length of Hospital Admission				Compliance With Medications			
Type of Childhood Adversity	Less Than 49 d *n* (%)	49 d or More *n* (%)	OR^a^ (95% CI)	*P*	Compliant *n* (%)	Not Compliant *n* (%)	OR^b^ (95% CI)	*P*
Parental separation								
No (*n* = 98)	59 (60.2)	39 (39.8)	—	—	61 (70.9)	25 (29.1)	—	—
Yes (*n* = 136)	59 (43.4)	77 (56.6)	**2.45** (1.06–5.66)	**.035**	69 (58.5)	49 (41.5)	**2.34** (1.11–4.92)	**.026**
Parental loss								
No (*n* = 207)	103 (49.7)	104 (50.2)	—	—	117 (64.6)	64 (35.4)	—	—
Yes (*n* = 26)	13 (50.0)	13 (50.0)	0.67 (0.19–2.35)	.536	12 (54.5)	10 (45.5)	1.18 (0.39–3.57)	.766
Physical abuse								
No (*n* = 178)	91 (51.1)	87 (48.8)	—	—	102 (64.5)	57 (35.8)	—	—
Yes (*n* = 58)	27 (46.5)	31 (53.4)	1.42 (0.51–4.00)	.504	30 (63.8)	17 (36.2)	1.23 (0.50–3.01)	.659
Sexual abuse								
No (*n* = 199)	99 (49.7)	100 (50.2)	—	—	118 (65.9)	61 (34.1)	—	—
Yes (*n* = 37)	19 (51.3)	18 (48.6)	0.74 (0.22–2.46)	.619	14 (51.9)	13 (48.1)	1.50 (0.51–4.35)	.458
Institutional care								
No (*n* = 224)	110 (49.1)	114 (50.9)	—	—	106 (66.7)	53 (33.3)	—	—
Yes (*n* = 12)	8 (66.7)	4 (33.3)	0.64 (0.10–4.16)	.637	21 (51.2)	20 (48.8)	1.25 (1.20–7.83)	.813
Family arrangements								
Up to 2 (*n* = 178)	89 (50.0)	89 (50.0)	—	—	106 (66.7)	53 (33.3)	—	—
3 or more (*n* = 48)	21 (43.7)	27 (56.2)	1.60 (0.55–4.71)	.390	21 (51.2)	20 (48.8)	2.67 (1.00–7.17)	.051
Total adversity								
0 (*n* = 66)	40 (60.6)	26 (39.4)	—	—	43 (74.1)	15 (25.9)	—	—
1 (*n* = 95)	47 (49.5)	48 (50.5)	2.36 (0.89–6.20)	.081	51 (58.6)	36 (41.4)	**2.81** (1.15–6.84)	**.023**
2 or more (*n* = 75)	31 (41.3)	44 (58.7)	2.28 (0.77–6.79)	.139	38 (62.3)	23 (37.7)	2.22 (0.82–6.05)	.117

*Note*: Figures in bold indicate *P* < .05.

^a^Adjusted for duration of untreated psychosis and baseline Global Assessment of Functioning symptom score.

^b^Adjusted for duration of untreated psychosis and baseline compliance with medication.

Evidence of a 2-fold increased odds of noncompliance with medications was found amongst those patients who reported childhood exposure to parental separation or disrupted family arrangements, though the latter association fell just short of statistical significance (*P* = .051). The association with compliance with medication at 1 year was similar for psychosis patients who reported single (OR = 2.81) and multiple (OR = 2.22) adverse childhood experiences.

## Discussion

To our knowledge, this is the first study systematically exploring the impact of different types of childhood adversity on 3 outcome domains over a 1-year period in first-presentation psychosis patients. Despite a high prevalence of childhood adversity in this sample (71%), compared to geographically matched controls (49%) reported in a previous study,^11^ we found no robust evidence that a history of adversity impacted on course of psychotic illness during the first year after presentation to services. This is inconsistent with previous findings that demonstrated associations between exposure to childhood abuse (sexual, physical, emotional)^16,18^ or parental loss^20,37^ and a more chronic course of illness.

However, in line with a previous study conducted on first-episode psychosis patients, in which childhood abuse was not associated with lack of symptomatic remission at follow-up,^22^ in the current study experiences of childhood adversity were not associated with lack of remission from psychotic symptoms in the 1-year follow-up period. There were also no differences between patients who reported most types of childhood adversity and those who did not report any childhood adversity in severity of symptoms at 1-year follow-up. The exception was for death of a parent before 17 years of age which was significantly associated with slightly less severe symptomatology at follow-up. Previous studies found an association between childhood abuse and more severe psychotic symptoms,^38,39^ though they were conducted on small samples (<100 patients) followed up to 6 months, and this makes comparison with the current study difficult.

In terms of social outcomes, psychosis patients reporting experiences of physical abuse in childhood were almost 3 times more likely to be single at follow-up compared to those patients that did not report this type of adversity, while no association was shown at baseline between the 2 subgroups (OR = 1.40, *P* = .360). Previous studies have shown that patients with psychosis who reported a history of childhood abuse had higher rates of avoidance and discomfort with closeness,^40^ and fewer of the psychological resources necessary for sustaining intimacy^41^ compared to those without such a history. Similar findings come from studies on adults with post-traumatic stress disorder^42,43^ that highlight an association between emotional distress and significant deficits in metacognition, namely the capacity of thinking about the thoughts and feelings of others.^44^ Attachment theory also suggests that early disruption of attachment in childhood can cause difficulties in source monitoring, emotion recognition, and the ability to form coherent representations of oneself and others,^45–47^ thus impairing the ability to initiate and maintain satisfying relationships in adulthood.^48,49^

No strong evidence for associations were found between types of childhood adversity and unemployment status at 1-year follow-up, though the ORs for physical abuse (OR = 1.67) and disrupted family arrangements (OR = 1.54) were suggestive of slightly higher proportions unemployed amongst these patients. Consistent with the current study, Conus et al^22^ found that a history of sexual and/or physical abuse amongst first-episode psychosis patients was not associated with unemployment. However, previous research has highlighted the link between childhood adversity and a higher rate of unemployment in patients with severe mental disorders over longer follow-up periods.^18,19^

In the current study, we also did not find robust associations between a history of childhood adversity and the global measure of social and vocational functioning at 1-year follow-up, with the exception of parental loss which, paradoxically, was associated with slightly better functioning. The negative results are largely in keeping with previous findings that first-episode psychosis patients exposed to sexual or physical abuse during childhood showed no differences in terms of functional outcome compared to nonexposed patients at 18-month follow-up.^22^ However, studies conducted in samples of chronic patients reported deficits in functioning in those reporting a history of adversity during childhood.^19,21,41,50,51^ This raises the possibility that the impact of some forms of childhood adversity may only be evident over longer follow-up periods or in those with more chronic forms of psychosis.

Finally, though, we did find that psychosis patients reporting a history of parental separation were more likely to spend longer on psychiatric wards and be noncompliant with medications 1 year after first contact with psychiatric services compared to those who did not report this childhood experience. Previous first-episode psychosis studies have also found significant associations between childhood adversity and longer stays in hospital,^23^ a higher number of admissions to hospital,^18^ and poor medication adherence.^17^ Moreover, individuals with traumatic childhood experiences have shown difficulties in seeking help and in maintaining relationships,^52,53^ especially with authority figures such as health professionals.^17,54^ Because of such difficulties, it might be challenging for mental health professionals to establish a good therapeutic alliance with patients with a history of parental separation^55^ and this, in turn, might prolong the time spent on a psychiatric ward and/or reduce compliance with treatments, including medication.

### Limitations

There are several limitations to this study. First, we asses sed childhood adversity using retrospective self-report. Although several studies have shown some bias in retrospective reports,^56^ such bias is considered insufficiently great to invalidate retrospective case-control studies of childhood experiences.^57^ Moreover, previous studies have demonstrated that the effect of childhood adversity on psychosis remains significant regardless of study design^2^ and histories of childhood adversity obtained by psychosis patients appear reliable over time and unaffected by current symptoms.^33,58^ Moreover, we utilized the CECA.Q to assess adversity which contains additional questions to obtain concrete details of the reported experiences and severity is determined by the researcher based on this additional information, thus reducing the subjectivity inherent in self-reports. We also attempted to enhance the validity of the self-reported experiences by scoring the severity of the responses in a standardized manner (see www.cecainterview.com), and using conservative cutoffs to ensure only severe adversity was considered in analyses. All of these factors increase the likelihood of an individual accurately remembering past adverse experiences.^57^ Given the low prevalence rate of psychotic disorders in the general population (approximately 3%),^59^ it would be very difficult to attempt to collect data on childhood adversity prospectively in a birth cohort as the sample size required to obtain a sufficient number of clinical cases would be too large to be cost-effective.

Second, although this was a fairly large first-presentation sample, the low prevalence of some forms of adversity is likely to have reduced our power to detect statistically significant associations with psychosis outcomes. This is indicated by reasonably wide CIs particularly for associations with being taken into care and parental loss. Therefore, our findings should be treated with appropriate caution and further research is required in larger epidemiological samples. It is also possible that the lack of impact on outcomes found for some forms of adversity was due to the length of delay between reported exposure to childhood adversity and subsequent onset and presentation to services for psychosis. However, the majority of our sample (81%) were aged 35 years or younger at presentation to services and only 2 cases were aged 60 or above, suggesting that for most patients this delay was not too long. Moreover, prospective studies have reported that effects of childhood adversity on mental and physical health outcomes persist over several decades.^60–62^

Our study failed to support a dose-response effect of childhood adversity on 1-year outcomes. These results might be an artifact of the approach we adopted to conceptualize and measure this dose-response effect. Schilling et al^63^ showed that the severity of childhood adversity experienced is more important in terms of later mental health outcomes than a simple cumulative adversity score. Similarly, Clausen and Crittenden^64^ argued that single instances of certain types of abuse (eg, physical or sexual) may be traumatic enough to produce detrimental effects while other adverse experiences may require repeated exposure to cause harm to the child. Therefore, if time had permitted, it would have been preferable to conduct a more in-depth interview, such as the full CECA interview,^65^ with participants to obtain more detailed information about their experiences and potentially improve the accuracy of reporting.^57^ This would allow investigation of the timing of exposure as well as relationship to perpetrators of childhood maltreatment and revictimization. Additionally, only specific types of adversity occurring during childhood were investigated in this study. Other forms of childhood adversity, such as bullying and exposure to domestic violence^66,67^ and other stressful life events in adulthood^68^ which have also been previously associated with psychosis, might demonstrate stronger associations with psychosis outcomes and confound the relationships found in the current study.

Another important limitation of this study is represented by selection and information bias arising from loss to follow-up and missing or inaccurate data. In an attempt to minimize attrition, the whereabouts or status of over 90% of the cohort was determined. Comparing those with and without some information available on course and outcome, there was no strong evidence of systematic bias. Although this does not entirely rule out selection bias, it does suggest attrition is unlikely to have seriously affected these findings. Nevertheless, the outcome data were obtained from clinical records rather than face-to-face interviews, thus limiting the type of outcomes that could be assessed. It is possible that periods of remission or information on overall clinical functioning were overestimated or underestimated as patients do not always disclose symptoms to clinicians and clinicians do not always accurately record what patients say. Additionally, many different healthcare professionals were involved in patient care, so the measurement of outcomes throughout the database would probably be less accurate and consistent than that achieved with a prospective cohort study design.^69^ However, clinical ratings were made by consensus after careful consideration of all available information and all efforts were made to rate the presence or absence of symptoms on the basis of clear and definite information. Bebbington et al^70^ also showed good reliability and validity of assessing remission and relapse in psychosis using case-notes.

Finally, duration of follow-up was relatively short, and it is possible that impact of trauma on outcome may become manifest only later and that 1-year follow-up may be accounted for by preexisting prognostic factors. Accordingly, the association between childhood adversities and clinical and social outcomes over 12 months has been corrected for the influence of several known baseline prognostic indicators, including DUP. Nonetheless, longer-term follow-up studies are required.

### Clinical Implications

Given the high prevalence of childhood adversities reported by first-presentation psychosis cases in this sample, routine assessment of adversity history and psychotherapies focused on adverse childhood experiences should be considered by services providing treatment to psychosis patients. Moreover, as shown in this study, without considering past exposure to (at least some) adverse experiences, the efforts to engage and treat psychosis patients may be unsuccessful.^71^ More research in this domain is therefore warranted, not only in order to better understand the mechanisms involved and direction of causality between adversity and its potential consequences but also to target psychological interventions to this complex issue.

## Funding

The work was supported by the Maudsley Charitable Fund; UK National Institute of Health Research (NIHR) Specialist Biomedical Research Centre for Mental Health grant (BRC–SLAM); UK Medical Research Council Population Health Scientist award (G1002366 to H.L.F.); MQ: Transforming Mental Health Fellows Award (MQ14F40 to H.L.F.); and Psychiatry Research Trust studentship (to A.T.). R.M.M. and A.S.D. receive salary support from the NIHR BRC.

## References

[1] Matheson SL Shepherd AM Pinchbeck RM Laurens KR Carr VJ Childhood adversity in schizophrenia: a systematic meta-analysis. Psychol Med. 2013;43:225–238. 22716913 10.1017/S0033291712000785

[2] Varese F Smeets F Drukker M Childhood adversities increase the risk of psychosis: a meta-analysis of patient-control, prospective- and cross-sectional cohort studies. Schizophr Bull. 2012;38:661–671. 22461484 10.1093/schbul/sbs050 PMC3406538

[3] Schäfer I Fisher HL Childhood trauma and psychosis - what is the evidence? Dialogues Clin Neurosci. 2011;13:360–365. 22033827 10.31887/DCNS.2011.13.2/ischaefer PMC3182006

[4] Arseneault L Cannon M Fisher HL Polanczyk G Moffitt TE Caspi A Childhood trauma and children’s emerging psychotic symptoms: A genetically sensitive longitudinal cohort study. Am J Psychiatry. 2011;168:65–72. 20952460 10.1176/appi.ajp.2010.10040567 PMC3536053

[5] Cutajar MC Mullen PE Ogloff JR Thomas SD Wells DL Spataro J Schizophrenia and other psychotic disorders in a cohort of sexually abused children. Arch Gen Psychiatry. 2010;67:1114–1119. 21041612 10.1001/archgenpsychiatry.2010.147

[6] De Loore E Drukker M Gunther N Childhood negative experiences and subclinical psychosis in adolescence: a longitudinal general population study. Early Interv Psychiatry. 2007;1:201–207.

[7] Janssen I Krabbendam L Bak M Childhood abuse as a risk factor for psychotic experiences. Acta Psychiatr Scand. 2004;109:38–45. 14674957 10.1046/j.0001-690x.2003.00217.x

[8] Kelleher I Keeley H Corcoran P Childhood trauma and psychosis in a prospective cohort study: cause, effect, and directionality. Am J Psychiatry. 2013;170:734–741. 23599019 10.1176/appi.ajp.2012.12091169

[9] Schreier A Wolke D Thomas K Prospective study of peer victimization in childhood and psychotic symptoms in a nonclinical population at age 12 years. Arch Gen Psychiatry. 2009;66:527–536. 19414712 10.1001/archgenpsychiatry.2009.23

[10] Spauwen J Krabbendam L Lieb R Wittchen HU van Os J Impact of psychological trauma on the development of psychotic symptoms: relationship with psychosis proneness. Br J Psychiatry. 2006;188:527–533. 16738342 10.1192/bjp.bp.105.011346

[11] Trotta A Di Forti M Iyegbe C Familial risk and childhood adversity interplay in the onset of psychosis. Br J Psychiatry Open. 2015;1:6–13. 10.1192/bjpo.bp.115.000158 PMC4995579 27703716

[12] Fisher HL Jones PB Fearon P The varying impact of type, timing and frequency of exposure to childhood adversity on its association with adult psychotic disorder. Psychol Med. 2010;40:1967–1978. 20178679 10.1017/S0033291710000231 PMC3272393

[13] Heins M Simons C Lataster T Childhood trauma and psychosis: a case-control and case-sibling comparison across different levels of genetic liability, psychopathology, and type of trauma. Am J Psychiatry. 2011;168:1286–1294. 21955935 10.1176/appi.ajp.2011.10101531

[14] Rubino IA Nanni RC Pozzi DM Siracusano A Early adverse experiences in schizophrenia and unipolar depression. J Nerv Ment Dis. 2009;197:65–68. 19155813 10.1097/NMD.0b013e3181925342

[15] Trotta A Murray RM Fisher HL The impact of childhood adversity on the persistence of psychotic symptoms: a systematic review and meta-analysis. Psychol Med. 2015;45:2481–2498. 25903153 10.1017/S0033291715000574

[16] Garno JL Goldberg JF Ramirez PM Ritzler BA Impact of childhood abuse on the clinical course of bipolar disorder. Br J Psychiatry. 2005;186:121–125. 15684234 10.1192/bjp.186.2.121

[17] Lecomte T Spidel A Leclerc C MacEwan GW Greaves C Bentall RP Predictors and profiles of treatment non-adherence and engagement in services problems in early psychosis. Schizophr Res. 2008;102:295–302. 18295458 10.1016/j.schres.2008.01.024

[18] Alvarez MJ Roura P Osés A Foguet Q Solà J Arrufat FX Prevalence and clinical impact of childhood trauma in patients with severe mental disorders. J Nerv Ment Dis. 2011;199:156–161. 21346485 10.1097/NMD.0b013e31820c751c

[19] Davidson G Shannon C Mulholland C Campbell J A longitudinal study of the effects of childhood trauma on symptoms and functioning of people with severe mental health problems. J Trauma Dissociation. 2009;10:57–68. 19197712 10.1080/15299730802485169

[20] Álvarez-Jiménez M Gleeson JF Henry LP Road to full recovery: longitudinal relationship between symptomatic remission and psychosocial recovery in first-episode psychosis over 7.5 years. Psychol Med. 2012;42:595–606. 21854682 10.1017/S0033291711001504

[21] Lysaker PH Nees MA Lancaster RS Davis LW Vocational function among persons with schizophrenia with and without history of childhood sexual trauma. J Trauma Stress. 2004;17:435–438. 15633923 10.1023/B:JOTS.0000048957.70768.b9

[22] Conus P Cotton S Schimmelmann BG McGorry PD Lambert M Pretreatment and outcome correlates of sexual and physical trauma in an epidemiological cohort of first-episode psychosis patients. Schizophr Bull. 2010;36:1105–1114. 19386579 10.1093/schbul/sbp009 PMC2963050

[23] Greenfield SF Strakowski SM Tohen M Batson SC Kolbrener ML Childhood abuse in first-episode psychosis. Br J Psychiatry. 1994;164:831–834. 7952992 10.1192/bjp.164.6.831

[24] Nanni V Uher R Danese A Childhood maltreatment predicts unfavorable course of illness and treatment outcome in depression: a meta-analysis. Am J Psychiatry. 2012;169:141–151. 22420036 10.1176/appi.ajp.2011.11020335

[25] Dilsaver SC Benazzi F Akiskal KK Posttraumatic stress disorder among adolescents with bipolar disorder and its relationship to suicidality. Bipolar Disord. 2007;9:649–655. 17845281 10.1111/j.1399-5618.2007.00396.x

[26] Leverich GS McElroy SL Suppes T Early physical and sexual abuse associated with an adverse course of bipolar illness. Biol Psychiatry. 2002;51:288–297. 11958779 10.1016/s0006-3223(01)01239-2

[27] World Health Organization. The ICD-10 Classification of Mental and Behavioural Disorders: Clinical Descriptions and Diagnostic Guidelines. Geneva, switzerland: World Health Organization; 1992.

[28] World Health Organization. Schedules for the Clinical Assessment of Neuropsychiatry. Geneva, switzerland: World Health Organisation; 1994.

[29] Singh SP Cooper JE Fisher HL Determining the chronology and components of psychosis onset: The Nottingham Onset Schedule (NOS). Schizophr Res. 2005;80:117–130. 15978778 10.1016/j.schres.2005.04.018

[30] Endicott J Spitzer RL Fleiss JL Cohen J The global assessment scale. A procedure for measuring overall severity of psychiatric disturbance. Arch Gen Psychiatry. 1976;33:766–771. 938196 10.1001/archpsyc.1976.01770060086012

[31] Bifulco A Bernazzani O Moran PM Jacobs C The childhood experience of care and abuse questionnaire (CECA.Q): validation in a community series. Br J Clin Psychol. 2005;44:563–581. 16368034 10.1348/014466505X35344

[32] Smith N Lam D Bifulco A Checkley S Childhood Experience of Care and Abuse Questionnaire (CECA.Q). Validation of a screening instrument for childhood adversity in clinical populations. Soc Psychiatry Psychiatr Epidemiol. 2002;37:572–579. 12545234 10.1007/s00127-002-0589-9

[33] Fisher HL Craig TK Fearon P Reliability and comparability of psychosis patients’ retrospective reports of childhood abuse. Schizophr Bull. 2011;37:546–553. 19776204 10.1093/schbul/sbp103 PMC3080697

[34] Sartorius N Jablensky A Korten A Early manifestations and first-contact incidence of schizophrenia in different cultures. A preliminary report on the initial evaluation phase of the WHO Collaborative Study on determinants of outcome of severe mental disorders. Psychol Med. 1986;16:909–928. 3493497 10.1017/s0033291700011910

[35] Jablensky A Sartorius N Ernberg G Schizophrenia: manifestations, incidence and course in different cultures. A World Health Organization ten-country study. Psychol Med Monogr Suppl. 1992;20:1–97. 1565705 10.1017/s0264180100000904

[36] Burnett R Mallett R Bhugra D Hutchinson G Der G Leff J The first contact of patients with schizophrenia with psychiatric services: social factors and pathways to care in a multi-ethnic population. Psychol Med. 1999;29:475–483. 10218939 10.1017/s0033291798008125

[37] Alvarez-Jimenez M Gleeson JF Henry LP Prediction of a single psychotic episode: a 7.5-year, prospective study in first-episode psychosis. Schizophr Res. 2011;125:236–246. 21081266 10.1016/j.schres.2010.10.020

[38] Lysaker PH Beattie NL Strasburger AM Davis LW Reported history of child sexual abuse in schizophrenia: associations with heightened symptom levels and poorer participation over four months in vocational rehabilitation. J Nerv Ment Dis. 2005;193:790–795. 16319700 10.1097/01.nmd.0000188970.11916.76

[39] Uçok A Bikmaz S The effects of childhood trauma in patients with first-episode schizophrenia. Acta Psychiatr Scand. 2007;116:371–377. 17919156 10.1111/j.1600-0447.2007.01079.x

[40] Couture S Lecomte T Leclerc C Personality characteristics and attachment in first episode psychosis: impact on social functioning. J Nerv Ment Dis. 2007;195:631–639. 17700294 10.1097/NMD.0b013e31811f4021

[41] Lysaker PH Meyer PS Evans JD Clements CA Marks KA Childhood sexual trauma and psychosocial functioning in adults with schizophrenia. Psychiatr Serv. 2001;52:1485–1488. 11684744 10.1176/appi.ps.52.11.1485

[42] Mazza M Giusti L Albanese A Mariano M Pino MC Roncone R Social cognition disorders in military police officers affected by posttraumatic stress disorder after the attack of An-Nasiriyah in Iraq 2006. Psychiatry Res. 2012;198:248–252. 22397917 10.1016/j.psychres.2011.11.027

[43] Nazarov A Frewen P Parlar M Theory of mind performance in women with posttraumatic stress disorder related to childhood abuse. Acta Psychiatr Scand. 2014;129:193–201. 23662597 10.1111/acps.12142

[44] Lysaker PH Vohs J Minor KS Metacognitive deficits in schizophrenia: Presence and associations with psychosocial outcomes. J Nerv Ment Dis. 2015;203:530–536. 26121151 10.1097/NMD.0000000000000323

[45] Fonagy P Gergely G Jurist EL Target M. Affect Regulation, Mentalization, and the Development of the Self. New York, NY: Other Press; 2002.

[46] Liotti G Prunetti E Metacognitive deficits in trauma related disorders: contingent on interpersonal motivational contexts? In: Dimaggio G Lysaker PH, eds. Metacognition and Severe Adult Mental Disorders: From Basic Research to Treatment. London, England: Routledge; 2010:196–214.

[47] Siegel DJ An interpersonal neurobiology of psychotherapy: the developing mind and the resolution of trauma. In: Solomon M Siegel DJ, eds. Healing trauma: Attachment, Mind, Body, and Brain. New York, NY: Norton; 2003:1–56.

[48] Klohnen EC Bera S Behavioural and experiential patterns of avoidantly and securely attached women across adulthood: A 30-year longitudinal perspective. J Pers Soc Psychol. 1998;74:230–250. 10.1037//0022-3514.74.1.211 9457783

[49] Swanson B Mallinckrodt B Family environment, love withdrawal, childhood sexual abuse, adult attachment. Psychother Res. 2001;11:455–472.

[50] Cusack KJ Frueh BC Brady KT Trauma history screening in a community mental health center. Psychiatr Serv. 2004;55:157–162. 14762240 10.1176/appi.ps.55.2.157

[51] Schenkel LS Spaulding WD DiLillo D Silverstein SM Histories of childhood maltreatment in schizophrenia: relationships with premorbid functioning, symptomatology, and cognitive deficits. Schizophr Res. 2005;76:273–286. 15949659 10.1016/j.schres.2005.03.003

[52] Berry K Barrowclough C Wearden A A review of the role of adult attachment style in psychosis: unexplored issues and questions for further research. Clin Psychol Rev. 2007;27:458–475. 17258365 10.1016/j.cpr.2006.09.006

[53] Pearlman LA Courtois CA Clinical applications of the attachment framework: Relational treatment of complex trauma. J Trauma Stress. 2005;18:449–459. 16281242 10.1002/jts.20052

[54] Mueser KT Rosenberg SD Goodman LA Trumbetta SL Trauma, PTSD, and the course of severe mental illness: an interactive model. Schizophr Res. 2002;53:123–143. 11728845 10.1016/s0920-9964(01)00173-6

[55] Goldsmith LP Lewis SW Dunn G Bentall RP Psychological treatments for early psychosis can be beneficial or harmful, depending on the therapeutic alliance: an instrumental variable analysis. Psychol Med. 2015;45:2365–2373. 25805118 10.1017/S003329171500032X PMC4501302

[56] Cohen P Cohen J The clinician’s illusion. Arch Gen Psychiatry. 1984;41:1178–1182. 6334503 10.1001/archpsyc.1984.01790230064010

[57] Hardt J Rutter M Validity of adult retrospective reports of adverse childhood experiences: review of the evidence. J Child Psychol Psychiatry. 2004;45:260–273. 14982240 10.1111/j.1469-7610.2004.00218.x

[58] Goodman LA Thompson KM Weinfurt K Reliability of reports of violent victimization and posttraumatic stress disorder among men and women with serious mental illness. J Trauma Stress. 1999;12:587–599. 10646178 10.1023/A:1024708916143

[59] van Os J Linscott RJ Myin-Germeys I Delespaul P Krabbendam L A systematic review and meta-analysis of the psychosis continuum: evidence for a psychosis proneness-persistence-impairment model of psychotic disorder. Psychol Med. 2009;39:179–195. 18606047 10.1017/S0033291708003814

[60] Rössler W Hengartner MP Ajdacic-Gross V Haker H Angst J Impact of childhood adversity on the onset and course of subclinical psychosis symptoms–results from a 30-year prospective community study. Schizophr Res. 2014;153:189–195. 24534797 10.1016/j.schres.2014.01.040

[61] Takizawa R Maughan B Arseneault L Adult health outcomes of childhood bullying victimization: evidence from a five-decade longitudinal British birth cohort. Am J Psychiatry. 2014;171:777–784. 24743774 10.1176/appi.ajp.2014.13101401

[62] Takizawa R Danese A Maughan B Arseneault L Bullying victimization in childhood predicts inflammation and obesity at mid-life: a five-decade birth cohort study. Psychol Med. 2015;doi:10.1017/S0033291715000653. 10.1017/S0033291715000653 25988703

[63] Schilling EA Aseltine RH Gore S The impact of cumulative childhood adversity on young adult mental health: measures, models, and interpretations. Soc Sci Med. 2008;66:1140–1151. 18177989 10.1016/j.socscimed.2007.11.023 PMC2362506

[64] Claussen AH Crittenden PM Physical and psychological maltreatment: relations among types of maltreatment. Child Abuse Negl. 1991;15:5–18. 2029672 10.1016/0145-2134(91)90085-r

[65] Bifulco A Brown GW Harris TO Childhood Experience of Care and Abuse (CECA): a retrospective interview measure. J Child Psychol Psychiatry. 1994;35:1419–1435. 7868637 10.1111/j.1469-7610.1994.tb01284.x

[66] Fisher HL Schreier A Zammit S Pathways between childhood victimization and psychosis-like symptoms in the ALSPAC birth cohort. Schizophr Bull. 2013;39:1045–1055. 22941743 10.1093/schbul/sbs088 PMC3756772

[67] Trotta A Di Forti M Mondelli V Prevalence of bullying victimisation amongst first-episode psychosis patients and unaffected controls. Schizophr Res. 2013;150:169–175. 23891482 10.1016/j.schres.2013.07.001 PMC3825661

[68] Beards S Gayer-Anderson C Borges S Fisher HL Morgan C Life events and psychosis: a review and meta-analysis. Schizophr Bull. 2013;39:740–747. 23671196 10.1093/schbul/sbt065 PMC3686461

[69] Sedgwick P Retrospective cohort studies: advantages and disadvantages. BMJ. 2014;348:g1072. 10.1136/bmj.g2979 25134102

[70] Bebbington PE Craig T Garety P Remission and relapse in psychosis: operational definitions based on case-note data. Psychol Med. 2006;36:1551–1562. 16911809 10.1017/S0033291706008579

[71] Grella CE Joshi V Treatment processes and outcomes among adolescents with a history of abuse who are in drug treatment. Child Maltreat. 2003;8:7–18. 12568501 10.1177/1077559502239610

